# Broken rice in a fermented total mixed ration improves carcass and marbling quality in fattened beef cattle

**DOI:** 10.5713/ajas.20.0288

**Published:** 2020-10-27

**Authors:** Salisa Kotupan, Kritapon Sommart

**Affiliations:** 1 Department of Animal Science, Faculty of Agriculture, Khon Kaen University, Khon Kaen 40002, Thailand

**Keywords:** Feeding, Growth Performances, Marbling, Rice, Ruminant

## Abstract

**Objective:**

This study aimed to determine the effects of replacing cassava chips with broken rice in a fermented total mixed ration diet on silage quality, feed intake, ruminal fermentation, growth performance, and carcass characteristics in the final phase of fattening beef cattle.

**Methods:**

Eighteen Charolais-Thai native crossbred steers (average initial body weight: 609.4±46 kg; average age 31.6 mo) were subjected to three *ad libitum* dietary regimes and were maintained in individual pens for 90 d before slaughter. The experimental design was a randomized complete block design by initial age and body weight with six replicates. The dietary regimens used different proportions of broken rice (0%, 16%, and 32% [w/w] of dry matter [DM]) instead of cassava chips in a fermented total mixed ration. All dietary treatments were evaluated for *in vitro* gas production and tested in *in vivo* feeding trials.

**Results:**

The *in vitro* experiments indicated that organic matter from broken rice was significantly more digestible than that from a cassava-based diet (p<0.05). Silage quality, nutrient intake, ruminal fermentation characteristics, carcass fat thickness, and marbling score substantially differed among treatments. The ruminal total volatile fatty acids, propionate concentration, dietary protein intake, and digestibility increased linearly (p<0.05) with broken rice, whereas acetate concentration and the acetate:propionate ratio decreased linearly (p<0.05) with broken rice (added up to 32 g/kg DM). Broken rice did not influence plasma metabolite levels or growth performance (p>0.05). However, the marbling score increased, and the carcass characteristics improved with broken rice.

**Conclusion:**

Substitution of cassava chips with broken rice in beef cattle diets may improve fattened beef carcass quality because broken rice increases rumen fermentation, fatty acid biosynthesis, and metabolic energy supply.

## INTRODUCTION

Farmers in Thailand frequently use fermented total mixed rations and total mixed ration silage as ruminant feeding systems. The number of agricultural cooperative service centers providing fermented total mixed ration feed is increasing. Fermented total mixed ration feeding technology combines the benefits of a nutrient-balanced diet of high-moisture agro-industrial by-products and homogeneous silage feed. In this process, high-moisture feeds such as cassava pulp, brewer’s grains, soybean curd residue, and vegetable residues are blended with dry feeds such as rice straw, rice bran, and oilseed cake [1–3]. Ensiled total mixed rations have superior storage life [1–3], aerobic stability [2,4], feed intake, and digestibility [5]. They enhance rumen fermentation characteristics, mitigate enteric methane [1], and improve growth performance [3,6], and the fatty acid composition of intramuscular fat (marbling) in beef cattle [7]. However, little research has been conducted to determine the effects of fermented total mixed ration feeding systems on the growth performance and carcass quality of beef cattle commercially fattened on this diet.

Marbling is associated with the tenderness of high-grade beef. It has a high market value in the urban consumer market wherein pricing is based on the marbling score. The productivity of fattened beef cattle has been low in developing tropical countries because of the genetic profiles of the animals and the provision of feeds and feeding systems based on rice straw and other low-quality agricultural by-products. Low feed quality has a negative impact on feed intake, digestion, and energy supply in fattened beef cattle. Consequently, it limits productivity and environmental sustainability [3,8,9]. Recent reports suggest that increases in the energy content of total mixed rations improve digestibility and growth performance in Brahmans, native Thai breeds, and Zebus crossbred with European beef cattle breeds [3,6,8]. For intramuscular fat deposition to occur in finishing cattle, the net energy consumed by the animals must exceed their requirements for maintenance and production. Hence, the degree of marbling is a function of the dietary energy supply [7,10,11]. The nutritional status of cattle is the main factor regulating their fatty acid biosynthesis. Acetate and propionate are volatile fatty acids (VFAs) produced during ruminal fermentation. They are the main energy precursors for fatty acid biosynthesis in ruminants [10]. Increasing the availability of ruminal fermentable starch by substituting Japanese brown rice for corn elevated the ruminal propionate and plasma glucose levels in lactating cows, thereby improving their milk fat yield and energy balance [12,13].

Broken rice (*Oryza sativa* L.; non-sticky; long-grained variety) is a by-products of agro-industrial rice mills. Approximately 5.2 million tons of it are produced annually in Thailand. Broken rice is suitable as an animal feed as it contains abundant nutrients, starch (that has a slow ruminal degradability rate), and 8% (w/w) crude protein. Thus, it is feasible as an alternative feed resource for high-producing beef cattle. In contrast, cassava has only 2% (w/w) crude protein [14]. When the proportion of Japanese brown rice with high ruminal degradation and fermentation rates was increased in the fermented total mixed ration-based diet fed to dairy cows, the relative ruminal propionic acid levels increased and the molar ratios of acetic to propionic acid decreased [12,13,15]. A recent study showed that replacing 20% of the corn grains with rice grains in the feed did not adversely affect rumen fermentation or growth performance in Hanwoo steers [16]. To the best of our knowledge, however, there is limited information about the impact of using broken rice instead of cassava chips as a primary energy feed source in fermented total mixed rations fed to fattening, marbled beef cattle in Thailand.

The objective of this study was to determine the effects of replacing cassava chips with broken rice in fermented total mixed rations on silage quality, feed intake, ruminal fermentation characteristics, growth performance, and carcass quality in the final phase of fattening Charolais-Thai native crossbred beef cattle.

## MATERIALS AND METHODS

### Experiment location and animal care

The experimental feeding trial was conducted at a commercial farm of the Nong Sung Agricultural Cooperative in Mukdahan Province, Thailand (latitude 16.50° N, longitude 104.35° E), between August 2018 and January 2019. All the procedures involving live animals were approved by the Animal Care and Use Committee of Khon Kaen University and were conducted in accordance with the published Ethics of Animal Experimentation of the National Research Council of Thailand (Record No. IACC-KKU-30/61, Reference No. 0514.1.75/8).

### Animals, diet, and experimental design

Eighteen Charolais-Thai native crossbred (50% *Bos taurus* ×50% *Bos indicus*) steers in the final phase of fattening with an average age of 31.6±2.7 mo and initial body weight of 609.4±46.9 kg were used in the feeding trial for a 90-d fattening phase before slaughtering. Each animal was treated for intestinal and external parasites (1 mL/50 kg body weight; Ivermectin and Clorsulon, Ivermectin F, Bangkok, Thailand) and vitamins A, D_3_, and E were intramuscularly injected to improve health (5 mL/head; Phenix, Bangkok, Thailand). The experimental animals underwent an adaptation period of 14 d before the 90-d feeding trial. The animals were housed in individual pens (3 m×4 m) with free access to food and drinking water throughout the experiment.

The individual animal in each pen was considered an experimental unit and was assigned to one of the six blocks (replicates) according to age and initial body weight using a randomized complete block design. Within each block, the animals were randomly assigned to one of three dietary treatments. The diets were offered *ad libitum*. The dietary treatments consisted of a diet containing either 32.0% cassava chips or 32.0% broken rice, or an equal mixture of cassava chips and broken rice (16%:16%) (Table 1). The experimental diet was formulated to meet the nutritional requirements of beef cattle [14] to produce a total mixed ration silage using ingredients such as rice straw, cassava chips, broken rice, palm meal, soybean meal, rice bran, cassava pulp, urea, minerals, and vitamins.

The fermented total mixed ration was prepared weekly in a vertical mixer with a 2,000 kg capacity (Pak Thong Chai Pasuset, Nakhon Ratchasima, Thailand). Approximately 1,000 kg of each batch of the treatment mixture was mixed and ensiled in plastic drums (150 L capacity, 48 cm diameter, 79 cm height), covered with a plastic lid, and stored at outdoor ambient temperature (25°C to 36°C) for at least 7 d.

### Sample collection and chemical analysis

Samples were taken before (day 0) and after (day 7) ensiling to evaluate the microbial counts, fermentation profiles, and nutritive values. The microorganisms were enumerated by the agar plate count method [17] and distinguished according to their colony and cell morphology. The counts were reported as colony forming units/g dry matter (DM) according to the procedure of Cao et al [17].

The *in vitro* gas production experiment followed the procedure of Sommart et al [18]. In brief, silage samples were dried in an oven at 55°C to a constant weight, milled, and passed through a 1-mm screen. Anaerobic techniques were used for all rumen fluid transfer and incubation steps. Two hundred milliliters rumen fluid was collected from each of the three cows before the morning feeding. The average age and weight of the animals were 5 yr and 466 kg, respectively. The rumen fluids were filtered through four layers of cheesecloth. Rumen inoculum medium was prepared by blending 660 mL rumen fluid and a buffer solution consisting of 1,095 mL distilled water, 730 mL buffer, 365 mL mineral macronutrients, 0.23 mL mineral micronutrients, 1 mL resazurine, and 60 mL reduction solution. Feed samples weighing ~0.5 g were placed in 50-mL serum bottles, which were then closed with rubber stoppers, crimp-sealed, injected with 40 mL rumen inoculum medium, and then incubated in a water bath at 39°C. Four replicated bottles were prepared to determine fermentation gas production and *in vitro* digestibility. For the first sample incubation set, the gas volumes were released from each bottle after 2 h, and every 2 h thereafter until 24, 48, 72, and 96 h incubation. Gas volumes were determined from the calibrated scale of the 20-mL glass syringe. After 24 h incubation, the second sample set was used for pH measurement with an electrode pH meter. *In vitro* dry and organic matter digestibility was also analyzed based on the procedure of Sommart et al [18].

Feed samples were collected weekly and pooled for chemical analysis. The DM content was determined by oven-drying the samples at 105°C to a constant weight. Subsamples were dried in an oven at 60°C to a constant weight, milled, passed through a 1-mm screen, and subjected to chemical analysis. Ash, crude protein, and ether extract was determined using Association of Official Analytical Chemists (AOAC) Methods 942.05, 984.13, and 920.39, respectively [19]. The organic matter content was calculated as the difference between the DM and the total ash, crude protein, and ether-extractable matter. Neutral and acid detergent fiber and lignin were measured with a fiber analyzer (ANKOM 200; ANKOM Technology, Macedon, NY, USA) according to the method of Van Soest et al [20]. Daily feed offered and feed refused were weighed and recorded for each animal. Daily feed and nutrient intake were calculated as the difference between the amount of feed offered and the amount of feed refused. Approximately 1 kg of animal feces was collected from each animal via anal stimulation in the morning for five consecutive days. Sample aliquots were thoroughly mixed and stored at −20°C until DM determination and chemical analysis. Digestibility was evaluated using acid-insoluble ash as a natural internal marker according the method of Van Keulen and Young [21].

Approximately 200 mL of rumen fluid was collected with an esophageal tube and a rumen stomach tube pump 3 h after the morning feeding. Ruminal pH was measured with a glass electrode pH meter (FiveGo; Mettler Toledo, Greifensee, Switzerland) immediately after sample collection. Ruminal fluids were separated from the feed particles through three layers of gauze, then 100 mL of rumen fluid was put into 150-mL plastic containers with 10 mL of 6 N HCL. These were collected and stored in ice buckets and transported to the laboratory. Rumen fluid was centrifuged at 13,000 rpm for 10 min at 4°C to determine VFA and lactic acid content using gas chromatography.

A blood sample was collected from each animal 3 h after the morning feeding at the end of the experimental period. Approximately 15 mL blood was collected from the jugular vein and placed in a sterilized vacuum tube (Greiner Bio-One [Thailand] Ltd., Chonburi, Thailand), packed on ice, and transported to the laboratory (Accreditation No. 4138/57; Khon Kaen TLC Lab Center Co. Ltd., Khon Kaen, Thailand) for plasma analyses. Plasma urea-N, glucose, triglyceride, cholesterol, total protein, and albumin concentrations were determined using the colorimetric method test kits (Roche Diagnostics, Indianapolis, IN, USA) and an automated analyzer (COBAS INTEGRA 400 plus analyzer, Roche Diagnostics, USA).

The animals were weighed at 08:30 at the start of the experiment and at 30, 60, and 90 d to assess growth performance. At the end of the experimental period, all animals were slaughtered, their carcass quality was evaluated, and their marbling scores were determined. Before slaughter, the animals were fasted with free water for ≥12 h and transported to the Nong Sung Agricultural Cooperative slaughterhouse. The carcass weights were recorded before and after the head, hide, feet, thoracic organs, internal fats, and abdominal organs were removed. The warm carcass weight and dressing percentage were calculated as the ratio of warm carcass weight to live weight. After dressing, the carcasses were transferred to an aging room at 4°C and chilled for 7 d. The chilled carcass weight was recorded, and the loin eye area was measured. The latter metric is the total area between the twelfth and thirteenth rib surfaces of the longissimus dorsi muscle. It was determined using a transfer and graph paper. Back fat thickness was measured at a point 3/4 of the longissimus dorsi muscle length at the twelfth rib. Marbling score (5 = very abundant; 1 = none) was assessed for the longissimus dorsi muscle between the twelfth and thirteenth ribs according to the Thai Agricultural Commodity and Food Standard.

### Statistical analysis

All data were subjected to analysis of variance using the generalized linear model procedure of SAS version 9.0 (SAS Institute Inc., Cary, NC, USA). The *in vitro* data were analyzed according to a spilt plot design arrangement in a completely randomized design with statistical modeling as follows:

(Equation 1) $${\text{Y}}_{\text{ij}}=\mu +\alpha_{\text{i}}+\delta \text{k}(\text{i})+\beta_{\text{j}}+\alpha \beta_{\text{ij}}+{ɛ}_{\text{ijk}},$$

where Y_ij_ is the dependent variable, μ is the overall mean, α_i_ is the effect of ensiling time (i = 1 to 2), β_j_ is the effect of dietary treatment (j = 1 to 3), αβ_ij_ is the effect of dietary× ensiling time, δk(i) is the main plot error, and ɛ_ijk_ is the residual error. Treatment means were considered statistically significant at p<0.05 using Duncan’s new multiple range test.

The *in vivo* data were analyzed according to a randomized complete block design, with the model included terms for treatment (df = 2) and block (df = 5) according to the following model:

(Equation 2) $${\text{Y}}_{\text{ij}}=\mu +\tau_{\text{i}}+\beta_{\text{j}}+{ɛ}_{\text{ij}},$$

where Y_ij_ is the dependent variable; μ is the overall mean, τ_i_ is the fixed effect of dietary treatment (i = 1 to 3), β_j_ is the fixed effect of block (j = 1 to 6), and ɛ_ij_ is the residual error. Treatment means were considered statistically significant at p<0.05 using Duncan’s new multiple range test. Linear and quadratic measures of orthogonal polynomial contrasts were estimated.

## RESULTS

### Diet characteristics

The chemical compositions of cassava chips, broken rice, and the various treatments are shown in Table 1. The fermentation metabolite profiles and microbial counts are shown in Table 2. The lactic acid bacterial counts were increased (p< 0.01), whereas those for the anaerobic and coliform bacteria and molds and yeasts were substantially reduced by day 7 of ensiling. The silage pH had also markedly decreased (p<0.01) by this time.

### *In vitro* gas production and digestibility

**Figure 1:**
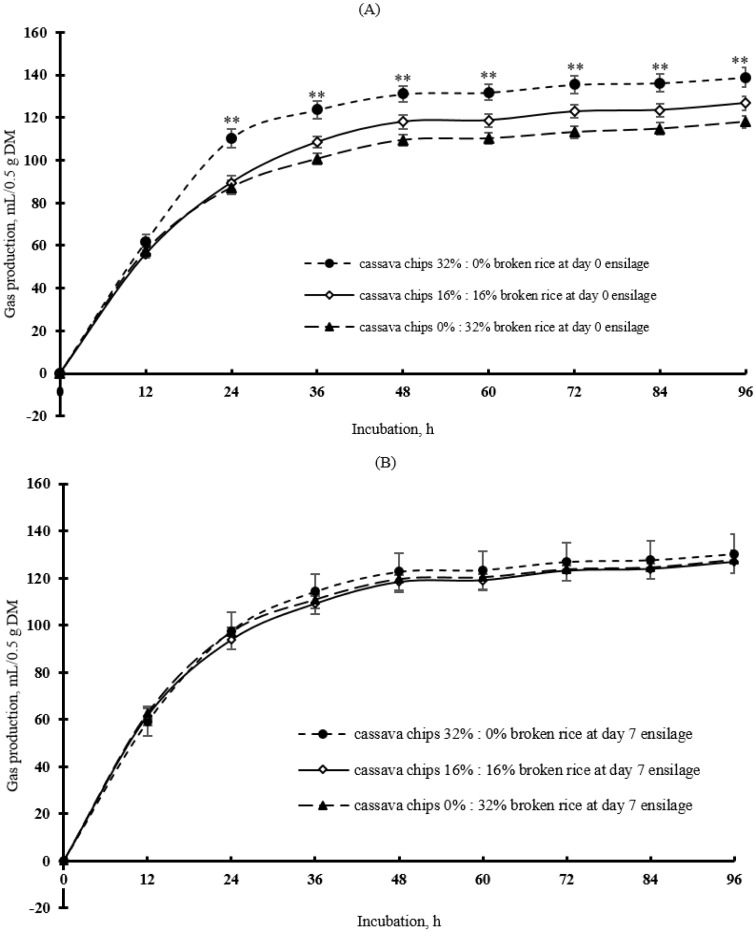
Cumulative gas production in response to various dietary treatments. (A) Day 0 silage. (B) Day 7 silage. Diets consisted of 32.0% (w/w) cassava chips, 32.0% (w/w) broken rice, or an equal mixture of cassava chips (16% w/w) and broken rice (16% w/w) in a fermented total mixed ration. For each time point, asterisks indicate at least one significant difference (** p<0.01) among treatments. Error bars represent standard deviation of n = 4 replicates.

The cumulative gas production rates under the various dietary treatments are shown in Figure 1. Total gas production and post-incubation rumen inoculum medium pH at 24 h and digestibility are shown in Table 3. Significant differences were observed among dietary treatments in terms of total gas production (p<0.01), rumen inoculum medium pH at 24 h (p<0.05), organic matter digestibility (p<0.04), and estimated metabolizable energy content (p<0.04) when the cassava chips were replaced with broken rice.

### Feed intake and digestibility

The feed intake and digestibility are listed in Table 4. An increase in the proportion of broken rice substituted for cassava chips in the diet showed a linear increase (p<0.01) in the daily protein intake, reflecting a higher crude protein content of broken rice. In contrast, there were significant linear decreases in the neutral and acid detergent fiber intake rates with the increased proportion of broken rice (p<0.05). The fresh, dry, organic matter content, and the ether extract intake did not differ among treatments (p>0.05).

There was a linear increase in crude protein digestibility with the proportion of broken rice in the diet (p<0.05). However, the apparent digestibility of dry and organic matter, ether extract, and neutral and acid detergent fiber did not differ among treatments (p>0.05).

### Ruminal fermentation characteristics and plasma metabolites

The average ruminal pH (6.7), ruminal ammonia-nitrogen level (4.6 mg/dL), and lactic acid concentration (0.3 mM) were not influenced by dietary treatment (Table 5). There were significant linear increases in the concentrations of ruminal total VFAs with the proportion of broken rice in the diet (p<0.01). In contrast, the acetate, butyrate, isobutyrate levels, and the acetate:propionate decreased with increasing proportion of broken rice in the diet (p<0.01). The propionate content increased linearly with the amount of broken rice in the diet (p<0.01). However, the dietary treatments had no significant influence on the rumen butyrate concentrations (p>0.05) or the plasma urea, glucose, triglyceride, cholesterol, total protein, or albumin levels (p>0.05).

### Growth performance and carcass characteristics

Growth performance and carcass characteristics are shown in Table 6. There were no significant differences among dietary treatments in terms of body weight gain, shrunk body weight, warm and chilled carcass weights and percentages, or loin eye area (p>0.05). Dietary treatment significantly influenced the marbling score according to a quadratic model (p<0.05). Back fat thickness and relative marbling percentage increased significantly with the proportion of broken rice (p<0.05).

## DISCUSSION

When broken rice replaced cassava chips in a fermented total mixed ration for beef cattle, overall production performance improved. Nutritive values, nutrient intake, rumen fermentation metabolite profiles, and the relative marbling score percentage were all improved.

Nutrient intake is a limiting factor for the energy supply required for animal maintenance and productivity. Tropical feeding systems often rely on low-quality feed sources that are deficient in nitrogen and digestible energy. Increasing the available energy enables animals to derive nutrients from carbohydrates, proteins, and fats. The resultant improvement in energy efficiency increases the ratio of energy intake to energy expenditure in the form of enteric methane emissions. Augmented energy retention in tropical beef cattle feeding systems was recently demonstrated [3,8]. Previous studies have reported relatively greater feed intake, digestibility, and nutrient availability in ruminants maintained on fermented total mixed rations than those administered non-fermented total mixed rations [5,7].

Fermented total mixed ration feeding technology has improved the nutrient balance, storage, and aerobic stability of agro-industrial by-products with a high moisture content. This management practice also has the advantage of furnishing homogeneous feed consisting of high-moisture products combined with dry feed silage [2,4,6]. The fermented total mixed ration is prepared by mixing wet by-products such as brewers’ grains, soybean curd residue, or cassava pulp with dry feedstuffs such as rice straw and oilseed cake and preserving this low-moisture diet as silage. Increasing the proportion of cassava pulp by replacing the rice straw conserves the nutritive values of the fermented total mixed ration, extends feed storage life, provides high aerobic stability, ameliorates rumen fermentation and digestibility, and mitigates enteric methane [1].

The fermented total mixed ration used in the current study produced a good fermentation metabolite profile. High-quality fermentation ensiling stabilized the nutrient content of the diets and was well preserved for >30 d; hence, it was cost-effective. The fermentation characteristics (Table 2) included a low pH within 7 d of ensiling. This finding was consistent with that of previous studies [3,6], wherein nutrient loss was detected during 90 d storage under a hot and humid tropical climate. In the present study, the ensiling period of ≥7 d was based on a previous study [1]. Seven days of ensiling resulted in good silage quality (pH 3.8 to 4.0; lactic acid content 63 to 66 g/kg DM; butyric acid content 0 to 0.03 g/kg DM; and NH_3_-N content 84 to 97 g/kg total N). An aerobic stability test indicated no self-heating within 30 h after opening. These properties are consistent with those previously reported [6,22,23]. Thus, silage should have a low pH, high lactic acid concentration, and only trace amounts of short chain VFAs. In the present study, the fermented total mixed ration was well preserved as its pH was low (3.8), and its lactic acid content was high (127.3 to 187.7 g/kg DM). A fermented total mixed ration feeding system preserves nutrients during storage under tropical conditions. The total lactic acid bacteria and the lactic acid concentration after 7 d were lower in the 0:32 treatment than they were in the 16:16 and 32:0 treatments. Therefore, the water-soluble carbohydrate content and the degradation rate were lowest for the 0:32 treatment. After 7 d of ensiling, the numbers of lactic acid and aerobic bacteria had increased and decreased, respectively. Coliforms and mycotoxin-forming molds were not detected, whereas the lactic acid-producing bacteria counts remained steady after 7 d of ensiling. Thus, the feed was free of any mycotoxin contamination.

The chemical compositions and the nutrient content (Table 1) of the diets containing cassava chips and broken rice differed slightly before and after ensiling of the total mixed ration. In this study, the change in chemical composition during ensiling resulted in a post-ensiling increase in protein content. This finding corroborated those of earlier studies [3,24]. The diets were formulated to have a crude protein content range of 9.1% to 11.0% before ensiling and to be iso-nitrogenous. The protein content rose to 10.3% to 11.5% after 7 d of ensiling. The relatively higher crude protein content in the fermented total mixed ration could be explained by the increase in the silage microbial population and the fermentation process over 7 d of ensiling under hot and humid tropical conditions. Post-ensiling decreases in neutral and acid detergent fiber content were observed. The non-fiber carbohydrate content increased after ensiling of the feeds containing comparatively lower proportions of cassava chips. Hence, lignocellulose may have been dissolved at the relatively low silage pH. The protein content increased with the proportion of broken rice in the diet as this material has a higher crude protein content than cassava chips. It was, therefore, expected that broken rice would increase nutrient availability and, by extension, the energy supply for the cattle. Gas production and digestibility (Table 3) significantly varied with the ratio of cassava to broken rice in the diet. A reduction in the ruminal fermentation rate at the gas phase may have enhanced the volatile fatty acid phase, especially in terms of propionic acid production. The latter augmented gluconeogenesis and, in turn, the energy supply for the host animal.

Rumen microorganisms provide energy sources to the host by transforming the glycogenic organic compounds in feed into usable fermentation end products and energy precursors such as short-chain fatty acids. In animals, the fat deposition rate is primarily controlled by the nutritional status, including the production of the VFAs, acetate and propionate, during ruminal fermentation. These substances are the main precursors for fatty acid biosynthesis in ruminants [10]. In the present study, with the increase in the proportion of broken rice in the fattened beef cattle diet, the ruminal fermentation metabolites consisted of significantly greater total VFA and propionic acid content, and a reduced acetic acid, iso-butyric acid content, and a lower acetic acid to propionic acid ratio than those of the animals maintained on diets with a low broken rice content (Table 5). Thus, increasing the proportion of broken rice in the feed elevated propionic acid production during rumen fermentation which, in turn, may have increased the relative percentage of marbling (50% of marbling score = 4). Previous studies [10,25,26] have suggested that propionate and lactate are precursors for intramuscular fatty acids in ruminants. Propionate and lactate are converted into acetyl coenzyme A, enter the tricarboxylic acid cycle, and improve the marbling score in fattened beef cattle. Ladeira et al [10] proposed that diets promoting ruminal propionate production and higher relative insulin and circulating glucose levels could increase intramuscular fat deposition in beef. Replacing corn with brown rice increased the availability of ruminal fermentable starch, ruminal propionate levels, and milk fat production [12,13]. Thus, a feed that increases propionate production and has high glycogenic and insulinogenic capacity (such as broken rice) could promote intramuscular fat deposition. The use of feed rich in broken rice might increase profitability and generate a product suitable for the premium beef market and consumers who prefer marbling (Table 6). In the Thai and global premium beef markets, wholesaler purchases are based on the marbling score (1 to 5 or another system) of the entire carcass. Traders and wholesalers manage butchering and meat quality grading in accordance with specific market requirements [27].

In the present study, only crude protein digestibility was influenced by dietary treatment. The protein in broken rice may have been more readily digested and absorbed than that in cassava chips by fattened beef cattle fed a fermented total mixed ration. The present study was restricted to short-term feeding during the final 90-d fattening phase before slaughter. Long-term investigations are required to develop practical and cost-effective guidelines for the incorporation of broken rice into fattened beef feeding systems.

In the present study, a commercial beef cattle fattening technology based on fermented total mixed rations was developed, comprising feed resources typically available in tropical countries. Replacing cassava chips with broken rice in a fermented total mixed ration at a rate of ≤32 g/kg DM improved feed efficiency and nutrient utilization. It increased the relative nutrient value, protein intake and digestibility, ruminal total VFAs, propionic acid concentration, and acetic acid to propionic acid ratio. This dietary modification had no apparent effect on plasma metabolite levels or growth performance. In addition, this feed amendment increased the marbling score percentage for fattened beef cattle.

## Figures and Tables

**Table 1:** Ingredients, feed cost, chemical composition, and metabolizable energy of experimental diets

Items	Cassava chips	Broken rice	Dietary treatment^1)^					

			Cassava chips:Broken rice					

			32:0	16:16	0:32	32:0	16:16	0:32
	
			Day 0 silage			Day 7 silage		
Ingredients (% DM)								
Rice straw	-	-	8.0	8.0	8.0	8.0	8.0	8.0
Cassava chips	-	-	32.0	16.0	0.0	32.0	16.0	0.0
Broken rice	-	-	0.0	16.0	32.0	0.0	16.0	32.0
Palm meal	-	-	4.0	4.0	4.0	4.0	4.0	4.0
Soybean meal	-	-	10.0	10.0	10.0	10.0	10.0	10.0
Rice bran	-	-	10.0	10.0	10.0	10.0	10.0	10.0
Cassava pulp	-	-	34.1	34.4	34.7	34.1	34.4	34.7
Urea	-	-	0.9	0.6	0.3	0.9	0.6	0.3
Mineral^2)^	-	-	0.5	0.5	0.5	0.5	0.5	0.5
Dicalcium phosphate	-	-	0.5	0.5	0.5	0.5	0.5	0.5
Total	-	-	100	100	100	100	100	100
Feed cost (TBH/kg FM)^3)^	7.20	12.30	3.08	3.46	3.83	3.08	3.46	3.83
Chemical composition (% DM)								
Dry matter	91.2	87.2	43.7	43.1	42.5	40.8	40.8	40.2
Organic matter	95.6	98.4	94.6	94.7	94.8	94.6	95.0	95.2
Crude protein	2.3	8.1	9.1	9.6	11.0	10.3	11.2	11.5
Ether extract	0.7	1.7	2.5	2.5	2.6	3.0	3.1	3.2
Neutral detergent fiber	10.3	6.8	33.9	33.7	33.4	28.4	27.1	26.4
Acid detergent fiber	7.2	0.6	25.3	21.8	21.7	18.6	18.0	17.7
Acid detergent lignin	0.4	0.1	4.3	3.4	3.4	2.9	2.8	2.6
Non-fiber carbohydrate^4)^	82.3	81.8	49.1	48.9	47.9	52.9	53.6	54.1
Metabolizable energy (MJ/kg DM)	12.6	12.7	12.8	12.8	12.9	13.1	13.2	13.3

DM, dry matter; FM, fresh matter.

1) Diets consisted of 32.0% (w/w) cassava chips, 32.0% (w/w) broken rice, or an equal mixture of cassava chips (16% w/w) and broken rice (16% w/w)

2) Minerals included 93.72 g Ca, 46.86 g P, 107.78 g Na, 18.55 g S, 8.24 g Mn, 7.49 g Zn, 3.37 g Mg, 1.17 g Cu, 0.15 g Co, 0.01 g K, 0.04 g I, and 0.02 g Se.

3) 1 US dollar = 35 TBH (Thai baht).

4) Calculated according to formula 100–(% crude protein+% ether extract+% ash+% neutral detergent fiber).

**Table 2:** Ensiling fermentation profile and microbial counts of various dietary treatments

Item	Day 0 silage^1)^			Day 7 silage^1)^			SEM	p-value	
	
	Cassava chips:Broken rice							Treatment	Day

	32:0	16:16	0:32	32:0	16:16	0:32			
pH	6.33	5.84	6.50	3.79	3.76	3.80	0.10	0.49	<0.01
Fermentation profile (g/kg DM)									
Lactic acid	NA	NA	NA	188^a^	170^b^	127^b^	23.4	0.01	ND
Acetic acid	NA	NA	NA	11.1^a^	10.4^a^	5.48^b^	0.68	0.03	ND
Propionic acid	NA	NA	NA	14.6^a^	8.04^b^	8.67^b^	0.37	0.02	ND
Butyric acid	NA	NA	NA	14.1	14.5	17.1	1.19	0.78	ND
Microbial counts (10^3^ CFU/g DM)									
Lactic acid bacteria	423^a^	335^b^	116^c^	1,200^a^	781^b^	123^c^	6.57	<0.01	<0.01
Aerobic bacteria	606^c^	1,514^b^	1,853^a^	73.5^a^	24.4^b^	10.7^c^	6.56	<0.01	<0.01
Coliform bacteria	19.2^a^	9.59^b^	5.33^c^	ND	ND	ND	2.56	<0.01	ND
Molds	0.60^a^	0.35^b^	0.35^b^	ND	ND	ND	3.20	<0.01	ND
Yeasts	32.0^a^	28.9^b^	5.91^c^	ND	ND	ND	2.05	<0.01	ND

SEM, standard error of the mean; DM, dry matter; NA, not available; ND, not detected; CFU, colony-forming units.

1) Diets consisted of 32.0% (w/w) cassava chips, 32.0% (w/w) broken rice, or an equal mixture of cassava chips (16% w/w) and broken rice (16% w/w).

a–c Mean values in same row within the same day with different superscripts differ significantly (p<0.05).

**Table 3:** *In vitro* cumulative gas volume and gas production kinetics, inoculum pH, digestibility, and estimation of metabolizable energy content in dietary treatments

Item	Day 0 silage^1)^			Day 7 silage^1)^			SEM	p-value	
	
	Cassava chips:Broken rice							Treatment	Day

	32:0	16:16	0:32	32:0	16:16	0:32			
Gas production (mL/0.5 g DM)									
24 h	110^a^	89.4^b^	87.2^b^	97.6^b^	93.8^b^	97.2^b^	1.45	<0.01	0.66
48 h	124^a^	109^b^	101^c^	114^b^	109^b^	111^b^	1.32	<0.01	0.71
72 h	135^a^	123^b^	114^c^	127^b^	123^b^	124^b^	1.38	<0.01	0.75
96 h	139^a^	127^b^	118^c^	130^b^	127^b^	128^b^	1.49	<0.01	0.79
Rumen inoculum									
pH 24 h	6.94^b^	6.94^b^	7.02^a^	6.94^b^	6.93^b^	7.03^a^	0.02	0.03	0.25
*In vitro* digestibility (%)									
IVDMD 24 h	75.2	76.8	78.1	78.7	79.6	80.4	1.34	0.50	0.14
IVOMD 24 h	91.3^b^	91.3^ab^	92.7^a^	93.8^b^	95.3^ab^	96.6^a^	0.51	0.04	0.02
ME (MJ/kg DM)	12.8^b^	12.8^ab^	12.9^a^	13.0^b^	13.2^ab^	13.3^a^	0.04	0.04	0.02

SEM, standard error of the mean; IVDMD, *in vitro* dry matter digestibility; IVOMD, *in vitro* organic matter digestibility; ME, metabolizable energy.

1) Diets consisted of 32.0% (w/w) cassava chips, 32.0% (w/w) broken rice, or an equal mixture of cassava chips (16% w/w) and broken rice (16% w/w).

2) Metabolizable energy (MJ/kg DM) = 5.1194+0.0845×(IVOMD 24 h).

a–c Mean values in same row and within the same day with different superscripts differ significantly (p<0.05).

**Table 4:** Daily nutrient intake and digestibility in Charolais-Thai native crossbred fattening beef cattle fed various dietary treatments (n = 6 per group)

Item	Cassava chips:Broken rice^1)^			SEM	p-value	Effects^2)^	
	
	32:0	16:16	0:32			L	Q
Nutrient intake							
Fresh matter (kg as fed/d)	15.8	15.9	15.8	0.18	0.85	0.92	0.59
Dry matter (kg DM/d)	6.43	6.50	6.35	0.08	0.43	0.38	0.34
Organic matter (kg DM/d)	5.07	5.06	5.03	0.06	0.56	0.32	0.77
Crude protein (kg DM d)	0.54^b^	0.59^a^	0.60^a^	0.01	<0.01	<0.01	0.01
Ether extract (kg DM/d)	0.15	0.15	0.16	<0.01	0.16	0.13	0.22
Neutral detergent fiber (kg DM/d)	1.24^a^	1.09^b^	1.08^b^	0.03	0.03	0.02	0.16
Acid detergent fiber (kg DM/d)	0.82^a^	0.74^b^	0.74^b^	0.02	0.05	0.04	0.17
Digestibility							
Dry matter (% DM)	88.0	87.1	88.8	0.80	0.38	0.49	0.24
Organic matter (% DM)	89.0	88.4	89.8	0.73	0.41	0.46	0.27
Crude protein (% DM)	86.1^b^	87.0^ab^	87.6^a^	0.34	0.04	0.02	0.65
Ether extract (% DM)	86.1	83.4	86.9	1.48	0.27	0.72	0.12
Neutral detergent fiber (% DM)	72.8	69.5	72.8	1.86	0.43	0.79	0.22
Acid detergent fiber (% DM)	68.7	65.6	68.8	2.25	0.54	0.97	0.28

DM, dry matter; SEM, standard error of the mean.

1) Diets consisted of 32.0% (w/w) cassava chips, 32.0% (w/w) broken rice, or an equal mixture of cassava chips (16% w/w) and broken rice (16% w/w).

2) Probability value of orthogonal polynomial contrast; L, linear; Q, quadratic.

a,b Mean values in same row with different superscripts differ significantly (p<0.05).

**Table 5:** Ruminal fermentation and blood plasma metabolites in Charolais-Thai native crossbred fattened beef cattle fed various dietary treatments (n = 6 per group)

Item	Cassava chips:Broken rice^1)^			SEM	p-value	Effects^2)^	
	
	32:0	16:16	0:32			L	Q
Ruminal fermentation characteristics							
pH	6.65	6.62	6.88	0.12	0.29	0.21	0.34
Ammonia-N (mg/dL)	5.31	4.20	4.38	0.70	0.48	0.36	0.43
Lactic acid (mM)	0.28	0.30	0.33	0.02	0.18	0.07	0.71
Total volatile fatty acid (mM)	122^b^	134^ab^	139^a^	4.32	0.01	<0.01	0.49
Acetic acid (%)	70.1^a^	68.4^a^	65.2^b^	0.78	<0.01	<0.01	0.38
Propionic acid (%)	15.6^c^	18.8^b^	21.0^a^	0.51	<0.01	<0.01	0.51
Iso-butyric acid (%)	1.00^a^	0.76^b^	0.80^b^	0.03	<0.01	<0.01	<0.01
Butyric acid (%)	9.88	9.68	9.16	0.31	0.31	0.15	0.63
Iso-valeric acid (%)	2.42^a^	1.27^b^	2.23^a^	0.22	<0.01	0.60	<0.01
Valeric acid (%)	1.02^b^	1.13^b^	1.64^a^	0.11	<0.01	0.01	0.15
Acetic:propionic acid ratio	4.62^a^	3.84^b^	3.26^c^	0.10	<0.01	<0.01	0.35
Plasma metabolites							
Urea-N (mg/dL)	5.55	5.78	5.01	0.47	0.49	0.42	0.39
Glucose (mg/dL)	67.8	68.2	69.3	2.29	0.89	0.65	0.88
Triglyceride (mg/dL)	34.5	24.7	35.0	5.24	0.33	0.95	0.15
Cholesterol (mg/dL)	136	134	142	10.2	0.86	0.69	0.72
Total protein (g/dL)	6.91	6.82	6.75	0.08	0.39	0.19	0.87
Albumin (g/dL)	3.93	3.76	3.75	0.09	0.32	0.18	0.51

SEM, standard error of the mean.

1) Diets consisted of 32.0% (w/w) cassava chips, 32.0% (w/w) broken rice, or an equal mixture of cassava chips (16% w/w) and broken rice (16% w/w).

2) Probability value of orthogonal polynomial contrast; L, linear; Q, quadratic.

a–c Mean values in same row with different superscripts differ significantly (p<0.05).

**Table 6:** Growth performance and carcass characteristics in Charolais-Thai native crossbred fattened beef cattle fed various dietary treatments (n = 6 per group)

Item	Cassava chips:Broken rice^1)^			SEM	p-value	Effects^2)^	
	
	32:0	16:16	0:32			L	Q
Growth performance							
Initial body weight (kg)	618	601	609	9.95	0.48	0.54	0.30
Final body weight (kg)	665	648	656	10.4	0.52	0.52	0.35
Body weight gain (kg)	47.0	47.4	46.2	8.62	0.99	0.95	0.94
Average daily gain (kg/d)	0.52	0.53	0.52	0.10	0.99	1.00	0.89
Carcass characteristics							
Warm carcass weight (kg)	405	388	387	8.70	0.32	0.19	0.46
Warm carcass (%)	57.8	57.3	57.1	1.22	0.91	0.68	0.92
Chilled carcass weight (kg)	396	378	380	8.36	0.29	0.20	0.34
Chilled carcass (%)	56.7	56.0	56.0	1.19	0.90	0.71	0.79
Loin eye area (cm^2^)	43.3	46.1	42.7	1.56	0.32	0.76	0.15
Back fat thickness (mm)	16.6 ^ab^	11.8 ^b^	17.8^a^	1.54	0.05	0.23	0.11
Marbling score (grade 1 to 5, %)	3.50^a^	2.83^b^	3.50^a^	0.19	0.05	1.00	0.02
Grade 1	0	0	0	-	-	-	-
Grade 2	0	16.7	0	-	-	-	-
Grade 3	66.6	83.3	50.0	-	-	-	-
Grade 4	33.4	0	50.0	-	-	-	-
Grade 5	0	0	0	-	-	-	-

SEM, standard error of the mean.

1) Diets consisted of 32.0% (w/w) cassava chips, 32.0% (w/w) broken rice, or an equal mixture of cassava chips (16% w/w) and broken rice (16% w/w).

2) Probability value of orthogonal polynomial contrast; L, linear; Q, quadratic.

a,b Mean values in same row with different superscripts differ significantly (p<0.05).
